# Low Level of Microsatellite Instability Correlates with Poor Clinical Prognosis in Stage II Colorectal Cancer Patients

**DOI:** 10.1155/2016/2196703

**Published:** 2016-06-27

**Authors:** Ehsan Nazemalhosseini Mojarad, Seyed Mohammad Hossein Kashfi, Hanieh Mirtalebi, Mohammad Yaghoob Taleghani, Pedram Azimzadeh, Sanaz Savabkar, Mohammad Amin Pourhoseingholi, Hasan Jalaeikhoo, Hamid Asadzadeh Aghdaei, Peter J. K. Kuppen, Mohammad Reza Zali

**Affiliations:** ^1^Basic and Molecular Epidemiology of Gastrointestinal Disorders Research Center, Research Institute for Gastroenterology and Liver Diseases, Shahid Beheshti University of Medical Sciences, Tehran, Iran; ^2^Gastroenterology and Liver Diseases Research Center, Research Institute for Gastroenterology and Liver Diseases, Shahid Beheshti University of Medical Sciences, Tehran, Iran; ^3^AJA Cancer Research Center (ACRC), AJA University of Medical Sciences, Tehran, Iran; ^4^Department of Surgery, Leiden University Medical Center, Leiden, Netherlands

## Abstract

The influence of microsatellite instability (MSI) on the prognosis of colorectal cancer (CRC) requires more investigation. We assessed the role of MSI status in survival of individuals diagnosed with primary colorectal cancer. In this retrospective cross-sectional study the MSI status was determined in 158 formalin-fixed paraffin-embedded tumors and their matched normal tissues from patients who underwent curative surgery. Cox proportional hazard modeling was performed to assess the clinical prognostic significance. In this study we found that MSI-H tumors were predominantly located in the colon versus rectum (*p* = 0.03), associated with poorer differentiation (*p* = 0.003) and TNM stage II/III of tumors (*p* = 0.02). In CRC patients with stage II, MSI-L cases showed significantly poorer survival compared with patients who had MSI-H or MSS tumors (*p* = 0.04). This study indicates that MSI-L tumors correlate with poorer clinical outcome in patients with stage II tumors (*p* = 0.04) or in tumors located in the colon (*p* = 0.02). MSI-L characterizes a distinct subgroup of CRC patients who have a poorer outcome. This study suggests that MSI status in CRC, as a clinical prognostic marker, is dependent on other factors, such as tumor stage and location.

## 1. Introduction

Colorectal cancer (CRC) develops either sporadically (85% of the cases), as part of a hereditary cancer syndrome (≤10%), or as a background of inflammatory bowel disease [1]. The incidence of CRC in Iran has been significantly increased since 1970 [2, 3]. According to Iran National Cancer Registry (INCR) report, approximately 51,000 new cases of cancers with mortality rate of 35,000 are identified every year. This makes cancer the third most common cause of mortality in Iran. In this regard, CRC is considered as the fourth most common cancer in Iranian population [4, 5]. The prevalence however is different in Iranian male and female gender. In this regard, CRC is the fourth most common cancer in Iranian male and the second in female [4, 6–9].

Two distinct pathways have been identified as main players in CRC: the microsatellite instability (MSI) and the chromosomal instability (CIN)/microsatellite stable (MSS) pathway [10]. Findings of these two pathways have led to the paradigm of CRC as a genetically heterogeneous disease. MSI pathway in hereditary and sporadic colorectal cancer occurs through two different mechanisms. In hereditary nonpolyposis colorectal cancer (HNPCC) the cause is a germline mutation in one of the DNA mismatch repair genes (*MLH1*,* MSH2*,* MSH6*, and* PMS2*), while MSI in sporadic colorectal cancer is predominantly due to hypermethylation of the* MLH1* promoter and sometimes sporadic mutations [11]. The DNA mismatch repair system works as a spell checker that identifies and then corrects mismatched base pairs in the DNA. Defects in the mismatch repair mechanisms lead to MSI status [1, 11, 12]. Currently, tumor stage is the most important predictor of prognosis for CRC patients. Implications of MSI in colorectal cancer continue to increase and many studies have evaluated the role of MSI test in clinical management [10–12]. Investigation of MSI status in CRC is warranted for three reasons: (a) as a potential screening tool for HNPCC, (b) as a potential predictive factor of chemotherapy response, and finally (c) as a prognostic marker [13]. The prognostic significance of MSI for patients with colorectal cancer is a subject of controversy. The mechanism by which MSI possibly influences clinical outcome is unknown. Based on the number of markers displaying MSI per tumor, three groups of tumors are defined: those with 30–40% of the markers showing instability (MSI-H); those with less than 30–40% of the markers showing instability (MSI-L), and those showing no instability (MSS) [14]. Some investigations reported that patients with MSI-H tumors have a better prognosis than those with MSI-L or MSS tumors [15, 16], whereas others reported that MSI in colorectal cancer was not an independent prognostic factor or had no prognostic significance in colorectal cancer [17, 18]. The biologic defect producing the low MSI (MSI-L) phenotype in colorectal cancer is poorly recognized; since there is no obvious biologic differentiation between MSI-L and microsatellite stable (MSS) colorectal cancer, these two phenotypes are generally merged when analyzed against the well-defined high MSI (MSI-H) phenotype. Recently, there is controversy in the definition of MSI-L because most tumors could be classified as MSI-L if an adequate number of markers are studied [19]. Several studies have demonstrated the prevalence of MSI among CRC patients in Iran [20–23]. However, information about the survival or mortality rate is unknown. In the present study, we evaluated the possible prognostic significance of MSI in colorectal cancer patients by determining the relationship between MSI status (MSI-H, MSI-L, and MSS) and prognosis in 158 patients who underwent resection for primary colorectal cancer.

## 2. Materials and Methods

In this study 158 consecutive CRC patients referred to Gastroenterology and Liver Diseases Research Center, Shahid Beheshti University of Medical Sciences, Tehran, Iran, from 2004 to 2010. The patients, who underwent surgical resection of adenocarcinoma of the colon or rectum and their characteristic and clinical data were available, were retrospectively included in this study. Informed consent was obtained from all patients or their relatives. Clinical information was recorded prospectively and registered in a database; this information included (i) age, sex, and personal and family medical history and (ii) tumor location, tumor differentiation, and TNM stage. Ethical approval for the study was obtained from the institutional review boards of the relevant centers.

### 2.1. MSI Analysis

Serial sections (5 *μ*m) from formalin-fixed paraffin-embedded matched normal and tumor tissues were routinely stained, and representative normal and tumor regions were identified by microscopic examination. Genomic DNA from each tumor and from corresponding normal tissue was purified using the QIAamp Tissue Kit (QIAGEN GmbH, Germany). Yield and purity of products were determined by electrophoresis on 0.8% agarose gel and spectrophotometry absorbance at 260 nm. Determination of MSI status was carried out using five mononucleotide repeat microsatellite targets (BAT25, BAT26, NR21, NR24, and NR27) using standard PCR techniques [24]. PCR products were denatured by electrophoresis on 5% denaturing polyacrylamide gels and were analyzed by an ABI 3130xl automated sequencer (Applied Biosystems, USA). Tumor samples that exhibited different allele peaks than the corresponding normal sample(s) were classified as MSI for that particular marker. MSI-H is defined when at least two of the five standard markers show instability in tumor DNA. MSI-L is defined when one MSI marker shows instability and others were microsatellite stable (MSS) when there was no instability detected on tumors. Analysis was performed twice if the results were ambiguous. The TNM (tumor, lymph nodes, and metastasis) staging system was applied to determine the severity of disease and the local or distant extent of disease spread. The TNM staging system of the American Joint Committee on Cancer (AJCC) is the preferred and standard staging system for CRC.

### 2.2. Statistical Analysis

Since the significance of MSI-L in CRC is poorly understood, phenotype of MSI was originally grouped into three levels including MSI-High, MSI-Low, and MS-Stable. Analysis was performed twice if the results were ambiguous. Statistical analysis was performed using the SPSS software program for Windows, Release 13.0.0 (SPSS Inc., Chicago, IL). Comparison of variables was performed using Pearson's Chi-square test, Fisher's exact test, or the Mann-Whitney *U* test, depending on the nature of the data. Two-tailed *p* < 0.05 was considered statistically significant. Colorectal cancer (CRC) overall survival was computed since the date of cancer diagnosis up to the date of death or end of follow-up (February, 2014). Patients who died due to causes which were unrelated to colorectal cancer were censored at the time of death and were excluded from the analyses. For survival analyses, the following variables were assessed: age, sex, location of the tumor (colon versus rectum), tumor-node-metastasis stage, and grade of differentiation (well/moderate versus poor), use of adjuvant therapy, age of diagnosis, family history and MSI. Overall survival analyses were done through a Cox proportional hazard function for both univariate and multivariate analyses and Kaplan-Meier (log-rank test) curves were plotted. Significance for all statistics were recorded if *p* < 0.05.

## 3. Results

### 3.1. Patients Descriptive

We identified 158 consecutive patients with colorectal cancer of whom pathological results and follow-up data were available. The clinicopathological features of patients enrolled in this study is present at Table 1. Of 158 samples analyzed, 76 were from males and 82 were from females subjects.

### 3.2. Microsatellite Instability

The subjects were subdivided into three groups by MSI testing: Thirty-five (22.2%) tumors were MSI-H, 21 MSI-L (13.3%) and 102 MSS (64.55%). Among 35 MSI-H tumors, instability of two markers was detected in 15 tumors (42.8%), 12 tumors (34.2%) had instability in three markers and 5 tumors (14.3%) had instability in four markers and 3 tumors (8.6%) were instable in all five mononucleotide markers. NR24 was the most instable marker (33 tumor) among MSI-H patients, However in 21 MSI-Low patients, the most instable marker was NR24 detected in 9 cases followed by Bat 25 (6 case), NR21 (3 case) and Bat26 (3 case). However none of MSI-L tumors were instable for NR27 mononucleotide marker.  Table 2. The clinicopathological characteristic of patients according to MSI status is shown in Table 3. The mean period of follow-up was 60.2 ± 24.5 months and the median period of follow-up were 53 months (range 2–120). Of 158 patients, 70 cases had family history of CRC and 88 did not have any history of CRC or other gastrointestinal cancers. According to our findings 29 patients had metastatic in the time of diagnosis and 129 were negative for metastatic status. Patients in the MSS group were found to be older than patients in the MSI-L and MSI-H groups (mean ages 53.16 years, 49.6 years and 50.32 years resp.). However this was not statistically significant. Interestingly, the high incidence of MSI-H (24/35, 68.6%) were presented in male gender and there was statistically significant relationship between MSI status and gender (*p* = 0.019). We found that MSI-H colorectal cancer were located predominantly in proximal colon sites versus rectal sites (*p* = 0.030), associated with poorer differentiation (*p* = 0.003), have a family history for GI cancer (*p* < 0.001), and showed less frequent systemic metastasis (*p* = 0.050) than MSI-L and MSS colorectal cancer. Distribution of tumor stages I–IV differed among patients with MSI-H, MSI-L and MSS tumors (*p* = 0.028).

### 3.3. Stage of CRC Correlation with Clinical Status

MSI-H distribution in stage III, II and I were 60.0%, 31.4% and 8.6% respectively. None of the 7 tumors with IV stage were shown to be MSI-H. We found no significant differences among patients with MSI-H, MSI-L and MSS tumors for T status, M status, vital status or adjuvant therapy, however the distribution of lymph node metastasis and age of diagnosis in MSI-H CRCs was statistically different with MSI-L and MSS tumors.

### 3.4. Univariate and Multivariate Analysis

To assess the effect of MSI status on survival, we used a Cox regression model for univariate and multivariate analysis. Among the prognostic variables for overall survival entered into univariate and multivariate analysis, all characteristic such as diagnostic age, tumor stage, adjuvant therapy, MSI status, tumor stage, and family history were not correlated with overall survival of patients (Table 4).

### 3.5. Survival

**Figure 1:**
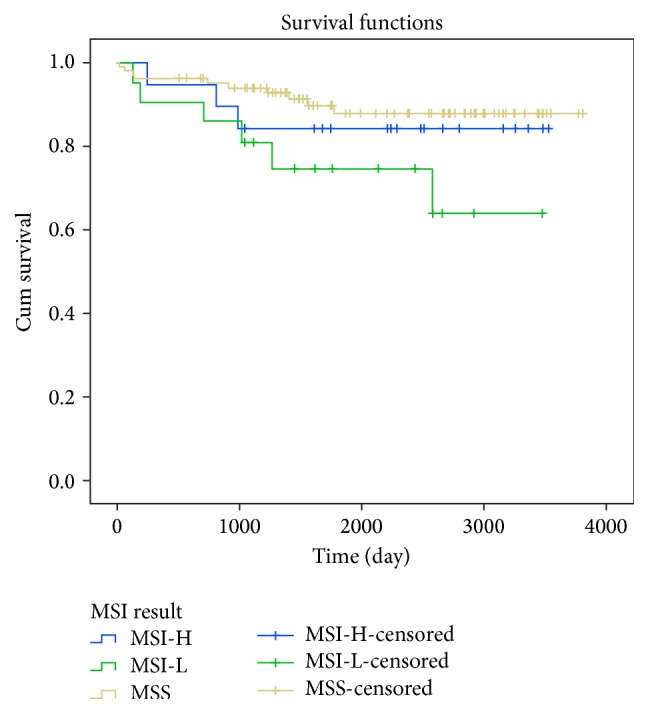
Kaplan-Meier curves of overall survival in colorectal cancer patients according to MSI status. Whereas the MSI-L tumors had poorer survival rate compared with MSI-H or MSS tumors, this result did not reach a significant rate, Log Rank *p* = 0.426.

**Figure 2:**
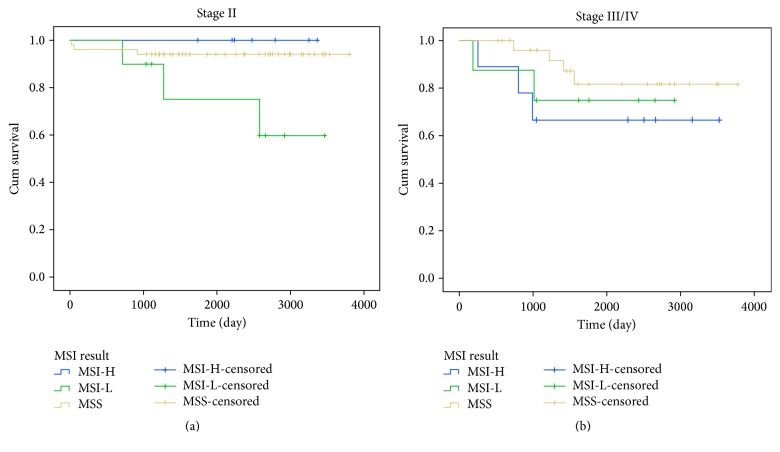
Kaplan-Meier curves of overall survival of patients according to MSI status stratified based on TNM stage. (a) Stage II colorectal cancer. Patient with MSI-L tumors showed significantly poorer survival compared with patients who had MSI-H or MSS tumors, Log Rank *p* = 0.048. (b) Stage III/IV colorectal cancer. There is no significant association between survival of patients in advanced stages with MSI status, Log Rank *p* = 0.430.

**Figure 3:**
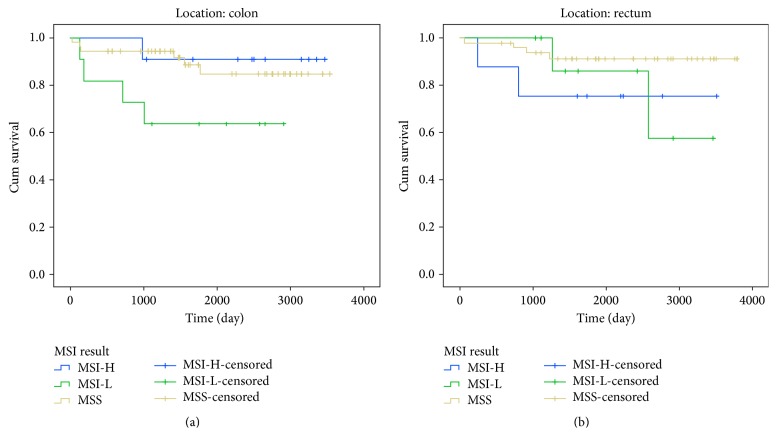
Kaplan-Meier curves of overall survival of patients according to MSI status stratified based on tumor location. (a) Colon. Patients with low MSI colon cancer show significantly poorer survival compared with patients who had MSI-H or MSS tumors, Log Rank *p* = 0.028. (b) Rectum. Patients with MSI-H rectal cancer showed poorer survival compared with patients who had MSI-L or MSS tumors, but this finding was not statically significant, Log Rank *p* = 0.180.

Overall survival curves relative to MSI status was obtained for all colorectal cancer patients presented in Figure 1. We did not observe clear influence of MSI status on overall survival in all colorectal cancer patients (*p* = 0.426). We also obtained Kaplan-Meier curves of overall survival in patients according to MSI status, stratified based on tumor location and TNM stages (Figures 2 and 3). Based on our results, in CRC Patients with stage I, MSI status is not considered as a valuable prognostic markers, Long Rank *p* = 0.742. Whereas in CRC Patients with stage II, MSI-L showed significantly poorer survival compared with patients who had MSI-H or MSS tumors, Long Rank *p* = 0.048, (Figure 2(a)). This indicates that MSI-L was clearly a marker of poorer prognosis in stage II colorectal cancers. However, we did not observe any significant association between overall survival of patients in advanced stages III/IV with MSI status, Long Rank *p* = 0.430, (Figure 2(b)). Kaplan-Meier curves of overall survival in patients according to MSI status stratified based on tumor location including colon and rectum are presented in Figures 3(a) and 3(b) respectively. According to our findings, patients with MS-L colon tumors, show significantly poorer survival compared with patients who had MSI-H or MSS tumors, Long Rank *p* = 0.028. But we did not observe the similar results in rectal tumors, Long Rank *p* = 0.180.

## 4. Discussion

Several epidemiological studies in Iran demonstrated that in comparison to western populations, the incidence of CRC is significantly higher in young Iranian patients and it has been reported that approximately 20% of all CRC cases occur in patients less than 40 years, whereas, this rate is extremely lower in western countries with 2 to 8% [25–30]. In addition to this high distribution of CRC prevalence in younger population of Iran in previous studies, in one study Azadeh et al. even reported a considerably higher proportion of 43% CRC incidence in patients less than 50 years old in Iranian population [27]. Although genetic factors have been proposed as one of the main risk factors contributed to this early onset of CRC in Iran, However, some studies indicated that this might be due to the high proportion of young population in Iran [6, 25]. In the present study we identified 35 MSI-H (22%), 21 MSI-L (13%), and 102 MSS (64%) tumors. MSI-H tumors were predominantly located in the colon than rectum (*p* = 0.03) and were associated with poorer differentiation (*p* = 0.003) and tumor, lymph nodes, and metastasis (TNM) stage II/III of tumors (*p* = 0.02) while MSI-L tumors were more frequent in patients aged younger than 50 years when diagnosed with colorectal cancer. The incidence of MSI observed in 158 colorectal cancer patients in the present study was similar to some previous reports from Iran [20–23], but it was also higher than other reports from other ethnic groups (5–20%) [15–17]. Ashktorab et al. documented a high incidence of MSI (45%) in CRC patients from African Americans population [31]. The incidence of MSI-L reported in this study was higher than another study in Iran [23]. In this study we used 5 mononucleotide markers to evaluate MSI status. Probably different sample sizes and different threshold markers were applied to assign MSI in each previous study, indicating this variety. In present study CRC patients with stage II MSI-L showed significantly poorer survival compared with patients who had MSI-H or MSS tumors (*p* = 0.04). This indicates that MSI-L was clearly a marker of poorer prognosis in stage II colorectal cancers. Our results documented distinct clinicopathologic characteristics of MSI-H, MSI-L, and MSS colorectal cancers. In line with other reports, most of the tumors in MSI-H were located in colon, poorly differentiated and frequent in stage III. High incidence of MSI-H in proximal located tumors in the colon was reported in many papers [32, 33]. However, Brim et al. have documented higher frequency of MSI-H tumors in distal colon in Omani patients [34]. The high incidence of MSI-H (24/35, 68.6%) was presented in male gender and there was statistically significant relationship between MSI status and gender (*p* = 0.01). In CRC patients with stage II, MSI-L cases showed significantly poorer survival compared with patients who had MSI-H or MSS tumors (*p* = 0.04). In contrast to several papers showing MSI is more common in women than in men [16, 17, 35]; we found a significant association between MSI status and male gender. The high prevalence of MSI-H in male was reported previously, but they did not find any significant association [15, 20]. The prevalence of MSI varied with tumor stage and was highest in stage III. This observation is in contrast to several other studies that showed the highest frequency of MSI in stage II [15–17, 20]. Similar to our finding, many other studies showed that the presence of distant metastases at the time of diagnosis (stage IV) is rare in the MSI subgroup of CRC [15, 20]. In addition, a relatively high frequency of patients diagnosed in age less than 50 was noted; we identified MSI-L tumors predominantly in this group age. The frequency rate of MSI-L in patients with age less than 40 was reported in a previous study in Iran [23], suggesting that this genetic pathway may play an important role in CRC development in Iranian patients between 40 and 50 years old. It has been reported that MSI-H colorectal tumors differ from MSI-L or MSS tumors in several pathological features [36–38] and MSI-L differ from MSS tumors, but the biological background of this feature is unknown [19, 39–43]. It was shown that MLH1 and MSH2 genes do not seem to be contributed to etiology of MSI-L [38]. Studies which indicated the* K-ras* mutations,* O* ^*6*^-*methylguanine DNA methyltransferase* (*MGMT*) promoter methylation, loss of* MGMT* gene expression, germline mutation of* hMSH6*, and a low level of allele loss near* APC* are associated with the MSI-L phenotype [19, 39–43]. Whereas several studies and two current meta-analyses suggested better prognosis and outcome for patients with MSI-H tumors [44–47], other studies could not confirm these findings [48, 49]. In line with the latter studies we observed no significant difference in overall survival between patients with MSS, MSI-L, and MSI-H tumors. In many prognostic studies, the MSI-L phenotype has not been considered as a separate category; this might be due to a lack of clear single marker for identification of this group of tumors. Our study has shown that MSI-L is associated with poorer survival in colorectal patients with stage II tumors. Many papers suggested that MSI status influences the prognosis of CRC only in specific stages [16, 19, 33, 35, 45]. Two recent studies documented poor prognosis of MSI-L in stage III colon cancer [19, 50]. Kohonen-Corish et al. showed that MSI-L characterizes a distinct subgroup of stage III colon cancer patients including the MSI-L subset of proximal colon cancer who have a poorer clinical outcome [19]. Nevertheless, there is now clear evidence that MSI-L tumors are a discrete molecular group on basis of gene expression data [41]. Together, these data suggest that MSI-L cancer may arise from a distinct carcinogenic pathway as compared to MSS and MSI-H CRC [51]. Other studies suggest that MSI-L and MSS have a common molecular background [52]. Though the biological basis of the good prognosis of patients with MSI-H is somewhat determined, the poor prognosis effect of MSI-L in colon cancer is unknown. It has been reported that the high mutational load in MSI-H tumors elicited a robust host immune response [53]. MSI-L and MSS have a high frequency of mutation of the p53 suppressor gene, explaining aggressive biological characteristics [37, 54]. Few studies have evaluated the effect of MSI status on rectal cancer survival [44, 55]. We assessed MSI status in relation to survival of individuals diagnosed with primary rectal cancer and primary colon cancer. We found poorer prognosis for MSI-L patients who had colon tumors and also poorer prognosis for MSI-H patients with rectum tumors, though this latter finding was not statically significant.

## 5. Conclusion

In conclusion, this is the first study in Iran to demonstrate the prognostic role of MSI in CRC patients. It seems that the clinical prognostic role of MSI status is dependent on stage and location of the tumor. Further studies with larger sample sizes are required to assess more precisely the impact of MSI-L on clinical outcome.

## Figures and Tables

**Table 1:** Clinicopathologic characteristics of patients enrolled in this study.

Variable		Total (*N*)
No patients		158

Gender	Female	82
	Male	76

Location of tumor	Colon	92
	Rectum	66

Differentiation	Well	64
	Moderately	51
	Poorly	39

TNM stage	I	21
	II	73
	III	57
	IV	7

MSI status	MSI-H	35
	MSL	21
	MSS	102

Family history	No^*∗*^	88
	Yes	70
	One or more FDR with CRC or adenoma	28
	One or more SDR with CRC or adenoma	17
	One or more SDR with HNPCC related cancers	25

Adjuvant therapy	Yes	39
	No	119

Vital status	Alive	141
	Deceased	17

Age of diagnosis	<50	89
	>50	69

Metastases	No	129
	Yes	29

Metastases location	Liver	14
	Ovary	6
	Other	9

^*∗*^No history of CRC, adenoma, or HNPCC related cancers.

**Table 2:** Frequency of instability in tumors according to pentaplex mononucleotide markers.

MSI status	Total	Marker				
		BAT-25	BAT-26	NR-21	NR-24	BAT-27
MSI-H	35	28 (80)	21 (60)	12 (34.3)	33 (91.4)	7 (20)
MSI-L	21	6 (12.6)	3 (6.3)	3 (6.3)	9 (18.9)	0
MSS	102	0	0	0	0	0

**Table 3:** Clinicopathological features of the study population according to MSI status.

		Total	MSI-H	MSI-L	MSS	*p* value
		*N*	*N* (%)	*N* (%)	*N* (%)	
Patients		158	35 (22.2)	21 (13.3)	102 (64.55)	

Mean age	Years		50.32	49.6	53.16	0.155

Gender	Female	82	11 (31.4)	11 (52.4)	60 (58.8)	0.019
	Male	76	24 (68.6)	10 (47.6)	42 (41.2)	

Location of tumor	Colon	92	27 (77.1)	11 (52.4)	54 (52.9)	0.030
	Rectum	66	8 (22.9)	10 (47.6)	48 (47.1)	

Differentiation	Well	64	8 (22.9)	9 (42.9)	47 (46.1)	0.003
	Moderately	51	8 (22.9)	7 (33.3)	36 (35.3)	
	Poorly	39	19 (54.3)	5 (23.8)	19 (18.6)	

TNM stage	I	21	3 (8.6)	2 (9.5)	16 (15.7)	0.028
	II	73	11 (31.4)	10 (47.6)	52 (51.0)	
	III	57	21 (60.0)	8 (38.1)	28 (27.5)	
	IV	7	—	1 (4.8)	6 (5.9)	

T stage	(T1 : T2 : T3 : T4)		4 (11.4) : 2 (5.7) : 29 (82.9) : —	1 (4.8) : 2 (9.5) : 15 (71.4) : 3 (14.3)	16 (15.7) : 11 (10.8) : 66 (64.7) : 9 (8.8)	0.104

N stage	(N0 : N1 : N2)		14 (40.0) : 17 (48.6) : 4 (11.4)	12 (57.1) : 7 (33.3) : 2 (9.5)	71 (69.6) : 25 (24.5) : 6 (5.9)	0.043

M stage	(M0 : M1)		35 (100) : —	20 (95.2) : 1 (4.8)	96 (94.1) : 6 (5.6)	0.162

Family history	Yes	70	24 (68.5)	9 (42.9)	37 (36.3)	<0.0001
	No	88	11 (31.4)	12 (57.1)	65 (63.7)	

Adjuvant therapy	Yes	39	2 (10.5)	7 (33.3)	30 (29.4)	0.054
	No	119	33 (94.3)	14 (66.7)	72 (70.6)	

Vital status	Living	141	32 (91.4)	17 (81.0)	92 (90.2)	0.463
	Deceased	17	3 (8.6)	4 (19.0)	10 (9.8)	

Age of diagnosis	<50	89	14 (40.0)	15 (71.4)	60 (58.8)	0.049
	>50	69	21 (60.0)	6 (26.6)	42 (41.2)	

Metastases	Yes	29	2 (5.7)	4 (19.0)	23 (22.5)	0.050
	No	129	33 (94.3)	17 (81.0)	79 (77.5)	

**Table 4:** Univariate and multivariate Cox regression analysis of possible prognostic variables and parameters that correlate with overall survival.

		Univariate analysis		Multivariate analysis	
		Hazard ratio for death (95% confidence interval)	*p* value	Hazard ratio for death (95% confidence interval)	*p* value
Gender	Female	1 ref.		1 ref.	
	Male	1.659 (0.630–4.368)	0.305	1.647 (0.524–5.180)	0.393

Location of tumor	Rectum	1 ref.		1 ref.	
	Colon	1.418 (0.524–3.836)	0.492	1.555 (0.480–5.031)	0.461

Differentiation	Well	1 ref.		1 ref.	
	Moderately	0.700 (0.234–2.091)	0.523	0.756 (0.221–2.587)	0.656
	Poorly	0.546 (0.147–2.031)	0.367	0.375 (0.076–1.840)	0.227

TNM stage	I	1 ref.		1 ref.	
	II	0.561 (0.103–3.065)	0.505	0.621 (0.097–3.969)	0.614
	III	0.758 (0.379–8.142)	0.471	3.848 (0.612–24.192)	0.151
	IV	4.277 (0.599–30.522)	0.147	10.087 (0.803–126.629)	0.073

Family history	No	1 ref.		1 ref.	
	Yes	0.672 (0.256–1.768)	0.421	0.746 (0.210–2.655)	0.651

Adjuvant therapy	No	1 ref.		1 ref.	
	Yes	1.224 (0.430–3.482)	0.705	0.274 (0.063–1.195)	0.085

MSI results	MSS	1 ref.		1 ref.	
	MSI-L	0.915 (0.251–3.336)	0.893	2.105 (0.584–7.580)	0.255
	MSH	2.026 (0.635–6.462)	0.233	0.512 (0.094–2.797)	0.440

Metastases	No	1 ref.		1 ref.	
	Yes	0.392 (0.145–1.061)	0.065	2.799 (0.707–11.084)	0.143

Age of diagnosis	<50	1 ref.		1 ref.	
	>50	0.723 (0.274–1.907)	0.512	2.240 (0.546–9.182)	0.263
